# Complete Penoscrotal Transposition with Other Extragenital Anomalies in a Neonate Delivered at Term

**DOI:** 10.1155/2021/6676301

**Published:** 2021-03-31

**Authors:** Ali Mahamudu Ayamba, Raymond Saa-Eru Maalman, Yaw Otchere Donkor, John Noah Anyorigiya

**Affiliations:** ^1^Department of Basic Medical Sciences, School of Medicine, University of Health and Allied Sciences, Ho, Volta Region, Ghana; ^2^Consultant Urologist, Department of Surgery, School of Medicine, University of Health and Allied Sciences, Ho, Volta Region, Ghana; ^3^Medical Officer, Margaret Maquart Catholic Hospital, Kpando, Volta Region, Ghana

## Abstract

A complete penoscrotal transposition (CPST) is a very uncommon congenital maldevelopment that is always associated with other abnormal body variations (malformations). We report a rare case of a term neonate delivered with CPST, which had a flaccid penis and an intact scrotum with unilateral limb and digital deformity, imperforate anus, cardiac malformation a facial deformity. Neonate died two hours after delivery. The foetal abnormality was not detected through routine antenatal services received by the mother.

## 1. Introduction

There is a rare occurrence of congenital scrotal anomalies categorized as penoscrotal transposition (PST), bifid scrotum, ectopic scrotum, and accessory scrotum [1]. This report describes the case of PST which is an uncommon external genitalia malformation present at birth where the penis is located inferior and posterior to the scrotum [1–3]. PST is generally associated with hypospadias and can either be described as complete or incomplete based on the location of the penis and scrotum [2]. Commonly reported form is the incomplete PST in which the penis is located in the middle of the scrotum, while in the complete PST, the penis that arises from the perineum covers completely by the scrotum [2].

The aetiology for the abnormal embryological sequence responsible for PST is still unclear [3, 4]. The genital tubercle and the labioscrotal swellings are the embryological origins of the penis and scrotum, respectively [5]. During normal embryonic development, in the 9th–11th week, the scrotal swellings migrate infero-medially and fuse in the midline caudal to the genital tubercle that forms the penis by the 12th week of gestation [4]. This is usually achieved under the influence of androgens and therefore PST arises from abnormal or arrested migration due to poor response to androgens [5]. Somoza et al. [4] and Park et al. [6] suggested that an abnormal positioning of the genital tubercle at the 6th gestation week concerning the scrotal swellings or a defective gubernaculum leads to PST.

Complete penoscrotal transposition (CPST) is frequently characterized by major and often life-threatening associated anomalies involving the urogenital, cardiovascular, gastrointestinal, and skeletal systems [4]. Common genital anomalies include hypospadias and chordee, and 100% of cases have a renal defect [5]. Several urogenital, cardiovascular, gastrointestinal, and skeletal system anomalies are, however, widely acknowledged associates, which very often present a major life-threatening situation in utero or in vivo. This suggests that penoscrotal transposition is often part of a wider group of embryonic malformations that should be investigated prenatally for an informed decision on the progress of pregnancy and route of delivery [7]. Such a case of CPST is described below.

## 2. Case Report

**Figure 1:**
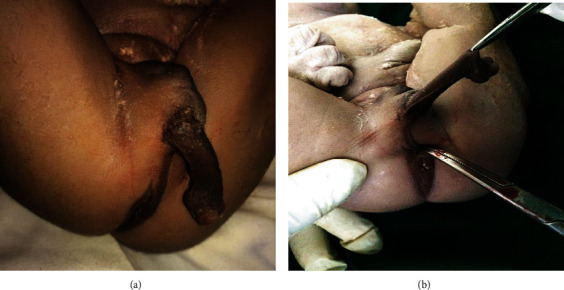
(a) Showing transposition of the hypoplastic penis and scrotum cephalically (b) showing the perineum and the root of the penis.

**Figure 2:**
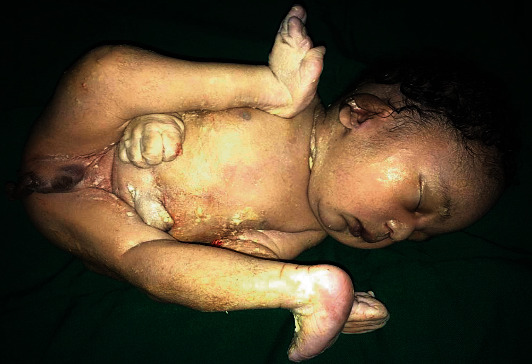
Lateral rotation of the left leg and 4 digits on the right foot.

We attended to a 21-year-old gravida one para zero woman who was referred to Margaret Marquart Catholic Hospital, Kpando, Ghana, on account of the prolonged first stage of labour with poor progress. A 2.4 kg newborn male was delivered at a gestation age of 41 weeks 3 days by a first-trimester scan and last menstrual period (LMP) via caesarean section on the account of nonassuring foetal heart rate. On physical examination, he was noticed to have a complete transposition of the external genitalia with cryptorchidism, hypoplastic penis from the perineum just above the blind anal position, and caudal to the scrotum (Figure 1(a)). Further examination revealed imperforate anus (Figure 1(b)), unilateral limb and digital deformity (Figure 2), and cardiac malformation and facial deformity.

The APGAR scores were 2 at the first and fifth minutes. Neonate died two hours after delivery. Other externally associated abnormalities included a laterally rotated left lower limb, which had four digits with a cleft between the third and fourth digits and low set ears. The mother had a booking visit to a hospital at the 11th-week gestation and a regular attendant for antenatal reviews at a primary care facility. Routine laboratory investigations during antenatal care (ANC) such as blood count indices, blood grouping, urinalysis, stool examination and microscopy, fasting blood sugar, retroviral status, and blood film for malaria parasites results were done and essentially normal. Record of the only ultrasound scan taken at 11^th^-week gestation did not indicate any anomaly. Throughout the pregnancy, she was on routine antenatal medications prescribed at the primary attending clinic, and she had no chronic illness. There was no evidence of the mother being exposed to teratogens during pregnancy.

The woman's 35-year-old husband has a 4-year-old daughter from a previous relationship who was delivered with an imperforate anus. History indicated that both parents of the 4-year-old daughter drink alcohol. Thus, the mother, a housewife, and the father, a driver of a local alcoholic beverage company, drink local alcoholic bitters with the intent to boost their appetite before and during the pregnancy period. However, the mother of the case being reported is a trader that do not drink alcohol. The 4-year-old daughter had a successful surgical reconstruction and currently living a normal life.

## 3. Discussion

Penoscrotal transposition (PST) is a congenital urogenital anomaly described first in 1923 by Appleby [7]. Its manifestations vary greatly from a slightly caudally displaced penis implanted within a bifid scrotum (incomplete) to an entirely transposed posterior penis originating from the perineum [5–7]. In this case report, there is a complete swap of position with cephalic scrotum and caudally located penis (Figures 1(a) and 1(b)). Also, there presents a hypoplastic penis located beneath the scrotum and directly cephalic to the blind anal position which makes it a complete penoscrotal transposition or a major type of external genital malposition as opposed to the commonly reported partial transposition and bifid scrotum.

CPST is a rare heterogeneous condition that could result from abnormal genital tubercle development around the 6th week of gestation. It could also be associated with a delay in the midline fusion of the urethral folds [8]. Human sex development is broadly divided into the genetic determination stage for which the presence of the Y-chromosome specify male, the stage of gonadal development (testes and ovaries), and the phenotypic stage which is largely influenced by specific hormonal secretion. The male external genitalia which differentiates during the phenotypic stage develops from a bipotential Anlagen, the genital tubercles, labioscrotal folds (urogenital folds and urogenital swellings). Up to the 9^th^ week, the external genitalia are indifferent in both sexes. Under the influence of androgens, the penis and scrotum develop and attain their final position with full differentiation of the organ at the end of the 12^th^ week. The embryological sequence responsible for malformations involving the genitourinary largely remains unclear.

Complex genitourinary positional anomalies are often associated with other systems abnormalities that may present as life-threatening during pregnancy, delivery, or postnatal.

Previous investigations have reported that anomalies associated with PST such as chordee and hypospadias could be found in 90% of patients, whereas imperforate anus, cardiologic, and gastrointestinal anomalies may be found in 30–60% of victims [5, 9].

The lessons we learnt from both the major and minor reported cases of varied combinations of these organ defects are a mostly functional and aesthetic outcome from surgery [4]; however, the perinatal outcomes are mixed [8]. The commonly associated anomalies noted in most reports include vertebral-, anal-, cardiac-, tracheoesophageal-, renal-, and limb (VACTERL) anomalies [5]. These congenital anomalies are conventionally viewed as compatible with intervention or incompatible with life. Surgical intervention may be challenging and worsening in resolving PST due to delay in performing the surgery to within age 12 months and 18 months. In order not to cause further harm to the penile, spongy is very delicate and great caution is needed to achieve a fully developed penis at puberty.

Clinical examination, prenatal obstetric imaging by skilled sonographers/radiologist using standard ultrasound scan machines as a start, and magnetic resonance machines or other modalities of investigations for the purpose enable the identification, classification of degree of anomaly, and monitoring of the progress of these pregnancies. This aims at offering intervention when necessary as well as planning for an appropriate and safe delivery route. Unfortunately, most antenatal clinics currently either lack these skilled labours, the equipment, or both in most developing countries including Ghana to detect organ and skeletal malformations.

## 4. Conclusion

A case of complete penoscrotal transposition was presented with major or life-threatening anomalies, which were not detected prenatally. This case illustrates the underutilisation of detection in utero as well as organ characterization, which is possible at the first trimester of pregnancy. It also informs us that there is much emphasis on maternal factors in the causes of congenital malformation, with a little investigation on the paternal contributory factors. In this case, the anomalies were incompatible with life and hence required psychological counselling to the parents.

It is, therefore, recommended that there is a need for pregnant women to have a complete skeletal scan before the end of the first trimester. There should also be in-depth psychological counselling on postdelivery trauma to parents of babies with such anomalies and perinatal death.
